# The Effect of Short and Long Term Endurance Training on Systemic, and Muscle and Prefrontal Cortex Tissue Oxygen Utilisation in 40 – 60 Year Old Women

**DOI:** 10.1371/journal.pone.0165433

**Published:** 2016-11-10

**Authors:** Gavin Buzza, Geoff P. Lovell, Christopher D. Askew, Hugo Kerhervé, Colin Solomon

**Affiliations:** 1 School of Health and Sport Sciences, University of the Sunshine Coast, Sippy Downs, Australia; 2 School of Social Sciences, University of the Sunshine Coast, Sippy Downs, Australia; Universidad Europea de Madrid, SPAIN

## Abstract

**Purpose:**

Aerobic endurance training (ET) increases systemic and peripheral oxygen utilisation over time, the adaptation pattern not being linear. However, the timing and mechanisms of changes in oxygen utilisation, associated with training beyond one year are not known. This study tested the hypothesis that in women aged 40–60 years performing the same current training load; systemic O_2_ utilisation (VO_2_) and tissue deoxyhaemoglobin (HHb) in the Vastus Lateralis (VL) and Gastrocnemius (GAST) would be higher in long term trained (LTT; > 5 yr) compared to a short term trained (STT; 6–24 months) participants during ramp incremental (RI) cycling, but similar during square-wave constant load (SWCL) cycling performed at the same relative intensity (below ventilatory turn point [VTP]); and that pre-frontal cortex (PFC) HHb would be similar between participant groups in both exercise conditions.

**Methods:**

Thirteen STT and 13 LTT participants performed RI and SWCL conditions on separate days. VO_2_, and VL, GAST, and PFC HHb were measured simultaneously.

**Results:**

VO_2peak_ was higher in LTT compared to STT, and VO_2_ was higher in LTT at each relative intensities of 25%, 80% and 90% of VTP in SWCL. HHb in the VL was significantly higher in LTT compared to STT at peak exercise (4.54 ± 3.82 vs 1.55 ± 2.33 μM), and at 25% (0.99 ± 1.43 vs 0.04 ± 0.96 μM), 80% (3.19 ± 2.93 vs 1.14 ± 1.82 μM) and 90% (4.62 ± 3.12 vs 2.07 ± 2.49 μM) of VTP in SWCL.

**Conclusions:**

The additional (12.9 ± 9.3) years of ET in LTT, resulted in higher VO_2_, and HHb in the VL at peak exercise, and sub—VTP exercise. These results indicate that in women 40–60 years old, systemic and muscle O_2_ utilisation continues to improve with ET beyond two years.

## Introduction

Irrespective of age and sex, in adults, there is a dose response relationship (to a limit) for increased duration and intensity of aerobic endurance training (ET) and improved maximum (peak) oxygen utilisation (VO_2peak_) [1, 2]. These adaptations are not linear, but rather follow a curvilinear increase [2]. Conversely, with advancing age, VO_2peak_ decreases in the same curvilinear pattern [3]. A meta-analysis indicated that in previously sedentary older adults, regular ET for 30–40 weeks elicits the largest improvements in VO_2peak_, with small improvements between 40–50 weeks, and a plateau at 50 weeks [2]. These results indicate that improvements in systemic O_2_ utilisation reach a plateau within one year of commencing regular ET, and only minimal increases will occur following prolonged (> 1 yr) ET.

In women over 60 years of age, physiological adaptations responsible for increased VO_2peak_ following ET vary depending on training history, these being peripheral [4–6], central [7, 8], or a combination of both [9]. Collectively, these previous results indicate that in older women, 1) regular ET decreases the typical age-related decline in VO_2_ through preserved central mechanisms; and 2), peripheral adaptations potentially only occur in individuals who start ET following a sedentary lifestyle. Peripheral adaptations play an important role in O_2_ utilisation, and ~90% of available O_2_ at peak exercise is consumed peripherally at the muscle mitochondria [10]. However, peripheral oxidative adaptations were not directly measured at the muscle in any of these studies, rather measuring arteriovenous oxygen difference which is calculated from central and systemic measurements. Therefore, the results did not directly indicate changes or differences in local muscle oxidative metabolism or O_2_ utilisation.

Peak VO_2_ and ventilatory turn point (VTP) are standard measures of systemic O_2_ utilisation and endurance performance [11–13]. During incremental exercise, tidal volume (V_T_) and breathing frequency (BF) increase simultaneously, then V_T_ plateaus, and further increases in pulmonary ventilation (V_E_) result from increases in BF only [14]. Ventilatory turn point corresponds to a metabolic rate where an increase in carbon dioxide production, relative to VO_2_, results in V_E_ increasing out of proportion to VO_2_ [15, 16]. Unlike VO_2peak_, VTP does not change (relative to VO_2peak_) with age [12, 13], and can increase following ET without concurrent improvements in VO_2peak_ [17]. Therefore, VO_2peak_ is likely to be higher in trained than untrained individuals; however, beyond 12–24 months of training, additional improvements may be seen in VTP, and not VO_2peak_.

Continuous wave near-infrared spectroscopy (NIRS) systems provide a more direct method of investigating changes in muscle and prefrontal cortex (PFC) deoxyhaemoglobin (HHb) during exercise [18, 19]. These systems have been used to compare muscle oxygenation (HHb, oxyhaemoglobin [O_2_Hb], total haemoglobin [tHb], and the tissue oxygenation index [TSI]) of trained and untrained young women [20–22] and ET adaptations in previously untrained [23] as well as highly trained young women [20, 22]. However, only one cross-sectional study of older women has reported the effect of endurance training on muscle HHb [21]. That study investigated the matching of O_2_ delivery to O_2_ utilisation [Δ tau (response time to a step increment in work load) HHb in the Vastus lateralis (VL) / Δ tau pulmonary VO_2_] of trained and untrained women aged 60–85 years during moderate exercise. The results indicated that trained, compared to untrained women had a better matching of O_2_ delivery to utilisation during moderate intensity exercise with a higher HHb amplitude reported in the trained compared to the untrained women at 90% VTP.

Changes in PFC oxygenation (HHb, O_2_Hb, tHb, TSI) occur during heavy exercise (in young men) [24–28], indicating an increased O_2_ utilisation and could be a potential mechanism for limiting exercise performance. Only two studies have measured PFC HHb in women during exercise [29, 30]. Neary et al. [29] reported significantly higher peak PFC HHb in a control group compared to those with chronic fatigue syndrome. Peltonen et al. [30] however, reported no difference in PFC HHb during ramp incremental (RI) exercise between healthy men and women. Further, although higher peak HHb levels have been reported in trained compared to untrained men [31], they were not significant. Therefore, it would be reasonable to expect that irrespective of the number of training years, PFC HHb would be similar in older women during moderate and high intensity exercise. Simultaneous measurements of VO_2_, and multiple muscle and PFC HHb could assist in determining a potential relationship between PFC HHb and exercise limitation [25].

Therefore the aim of this study was to determine the difference in systemic O_2_ utilisation (VO_2_) and multiple local muscle and PFC HHb between short term (STT; 6–24 months) and long term (LTT; > 5 years) endurance trained women aged 40–60 years matched for current training load, during two different cycling exercise conditions, RI peak and sub-maximal square wave (SWCL). It was hypothesised that; 1) VO_2_ would be higher in LTT compared to STT at VTP and VO_2peak_ during RI cycling, but similar at the same relative intensity (25%, 80% and 90% VTP) during SWCL cycling, and 2) muscle HHb would be higher in LTT compared to the STT in the vastus lateralis at VTP and peak exercise during RI cycling, but similar at the same relative intensities (25%, 80% and 90% VTP) during SWCL cycling, and that there would be no difference in PFC HHb between the groups at any exercise intensity.

## Methods

### Ethical Approval

This study was approved by the Human Research Ethics Committee at the University of the Sunshine Coast (S/14/676) and participants provided written informed consent.

### Study Design

The study used a cross-sectional, two group, repeated measures design. The independent variables were age, current training load and years of training. The dependent variables were systemic O_2_ utilisation (VO_2_), deoxygenated haemoglobin (HHb), heart rate (HR) and rating of perceived exertion (RPE) (modified Borg 1–10 scale) [32].

Each participant attended two testing sessions in a temperature controlled (20–23°C) exercise physiology laboratory. Prior to each session, participants abstained from alcohol and intense exercise for 24 hours and food and caffeine for four hours. Timing with the menstrual cycle was not controlled for as menstrual cycle phases have no significant effect on VTP [33] or VO_2peak_ [34–36].

### Participants

The two groups of older aerobically trained Caucasian women consisted of one group of 13 short term trained (STT) women, having regularly performed > 150 minutes of moderate to vigorous exercise per week (including cycling) over the last six to 24 months, and one group of 13 long term trained (LTT) women, having regularly performed > 150 minutes of moderate to vigorous exercise per week (including cycling) for at least the last five years. All participants were actively training for a minimum of 11 months every year. Current average ET load (time x intensity) was the same between groups, but as expected by design, lifetime ET was significantly less in STT compared to the LTT (p < 0.05). The participants’ physical characteristics and training history are provided in Table 1. Medical screening was conducted using the Physical Activity Readiness Questionnaire [37] and a Medical Health Questionnaire. Exclusion criteria were any cardiovascular, respiratory, metabolic and musculoskeletal disease, any health related issues or medications that would compromise participant safety and or impact exercise capacity or O_2_ utilisation. Training status was determined using self-reported physical activity training logs. Current training years was calculated as the number of continuous years of ET meeting the criteria outlined above. Current training load was determined by adding the product of each training session duration (in minutes) and intensity (1 = low, 2 = moderate and 3 = high) over seven days.

**Table 1:** Participant characteristics for short term trained and long term trained older women.

Characteristic	STT	LTT
Age (yr)	51.5 (5.0)	47.5 (5.0)
Weight (kg)	65.9 (10.5)	63.2 (7.4)
Height (cm)	164.4 (4.7)	167.5 (2.0)
LVL adipose (thickness)	12.8 (3.7)	9.8 (2.8)*
LGAS adipose (thickness)	13.1 (3.6)	10.1 (2.5)*
Current training (yr)	1.6 (0.5)	14.5 (9.8) *
Lifetime training (yr)	4.9 (3.8)	16.1 (8.1) *
Average weekly training load	862.8 (190.5)	987.6 (274.8)
VTP VO_2_ (mL · kg^-1.^ min^-1^)	20.2 (5.1)	29.0 (6.4) *
VTP % of Peak (mL · kg^-1.^ min^-1^)	65.6 (9.1)	70.7 (5.7)
VTP VO_2_ (L · min^-1^)	1.5 (0.4)	1.9 (0.4) *
VTP % of Peak (L · min^-1^)	72.0 (7.4)	75.3 (8.9)

Values are mean (SD).

* Significant difference between groups p = < 0.05.

Average weekly training load = minutes x intensity (light = 1, moderate = 2 and high = 3).

STT: Short Term Trained; LTT: Long Term Trained; LVL: Left Vastus Lateralis; LGAS: Left Gastrocnemius; VTP: Ventilatory Turn point.

### Testing Sessions

#### Session One

The aim of session one was to determine VO_2_ and HHb values at VTP and peak exercise. Anthropometric (height, mass, and thigh and calf skinfold [Harpenden skinfold calipers, British Indicators Ltd, UK]) and pulmonary function (Spiro II spirometer, Medical International Research, Rome, Italy) data were recorded. Adipose tissue greater than half the distance between the NIRS source and detector (< 17.5 mm) can affect the NIRS signal [38]. No participants were excluded following pulmonary function or skinfold thickness tests.

Heart rate and NIRS detectors were fitted and participants were instructed on the RPE scale. While the participant was seated on the bike, five minutes of resting measures were recorded, the last minute included VO_2_ measures. Participants then performed a ramp incremental test (1 W every 3 s) to volitational cessation, on a Velotron cycle ergometer (Racermate, Seattle, USA). During the test, VO_2_, HR and HHb were recorded continuously, while RPE was recorded during the last 10 s of each minute.

#### Session Two

Session two was conducted three to 28 days after session one. The aim of this session was to determine VO_2_ and HHb during exercise at the same relative intensity below VTP. Following the same preparation and procedures used in the RI test, participants completed a SWCL cycling protocol where the intensity was set as a percentage of VTP. The timing and designed percentages were; three minutes at 25%, 80%, 25%, 20 minutes at 90% and three minutes at 25%.

During the test, VO_2_, HR and HHb were recorded continuously, while RPE was recorded during the last 10 seconds of the third minute of each of the three minute SWCL stages, and every forth minute during the 20 minute stage. No feedback or encouragement was provided during this test to minimise cognitive stimulus.

### Measurements

#### Systemic Oxygen Utilisation and Ventilation

Expired gas analysis (Parvo Medics, Sandy UT, USA) was used for the determination of ventilation, tidal volume and breathing frequency, oxygen consumption (VO_2_), carbon dioxide production (VCO_2_), and respiratory exchange ratio during exercise and is presented in absolute (L^.^ min^-1^) and relative (mL^.^ kg^-1.^ min^-1^) values. The VO_2peak_ was determined as the highest 15 second average VO_2_ value during the last minute of the RI exercise test.

#### Ventilatory Turn Point

Ventilatory turn point was determined using the V-slope method [8, 39, 40]. Briefly, visual inspection determined the VO_2_ at which CO_2_ output (VCO_2_) increased out of proportion in relation to VO_2_ with an increase in the ratio of minute ventilation to VO_2_ ratio. This point was time matched with the Power (in Watts) of the cycle ergometer.

#### Heart Rate and Rating of Perceived Exertion

During both exercise conditions, HR was recorded continuously (Polar Electro, Kempele, Finland). Maximal heart rate was recorded as the highest HR obtained during the same 15 second period as that used for the VO_2peak_, which was the highest HR for each participant.

The participant’s rating of perceived exertion was measured using Borg’s 1–10 Category-Ratio (CR-10) ratings of perceived exertion scale.

#### Tissue Deoxyhaemoglobin

Local tissue oxygenation (HHb, O_2_Hb, tHb and TSI) data were measured continuously and simultaneously from the left and right VL, the left GAST and the left PFC with a multi-channel NIRS system (PortaMon and Portalite, Artinis Medical Systems BV, Zetten, Netherlands). Muscle optodes were placed over the middle of the muscle belly, fixed using adhesive tape and wrapped with low compression black elastic bandage (to prevent movement and extraneous light). For PFC monitoring, the optode was placed 1–2 cm over the left PFC above the eyebrow as used in previous studies [41, 42], fixed using adhesive tape and covered with a black headband. Differential pathway factor (DPF) depends on the optical characteristics of tissue [43] and depending on age, the path length of the photons is 4–6.5 times longer than the spacing between the optodes. To account for age a formula (DPF = 4.99 + 0.067[Age^0.814^]) was applied. This formula derived from data from Duncan et al. [44] is validated for ages 17–50 years. For all participants over the age of 50 the DPF range was set at 50 years of age.

**Fig 1:**
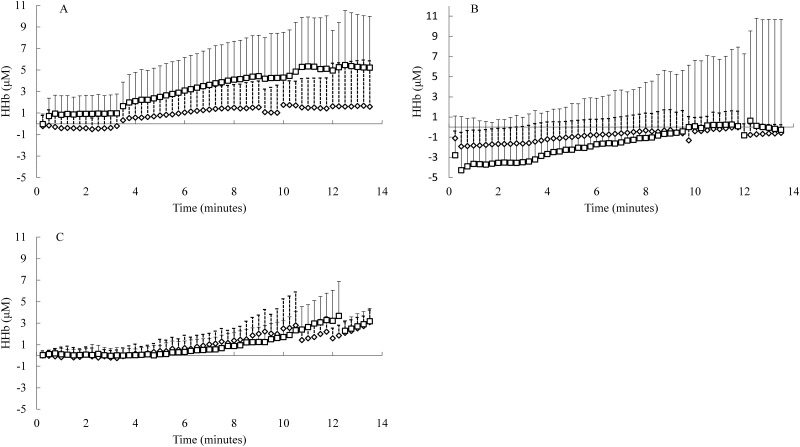
Ramp Incremental (cycling); deoxygenated haemoglobin HHb measures. Panel (A) HHb in the LVL. Panel (B) HHb in the GAST. Panel (C) HHb in the PFC. STT 0–8 min (n = 13); 8–9.5 min (n = 7–11); 9.5–13.5 min (n = 2–5). LTT 0–9 min (n = 13); 9–12 (n = 5–12); 12–14 (n = 3–5).

**Fig 2:**
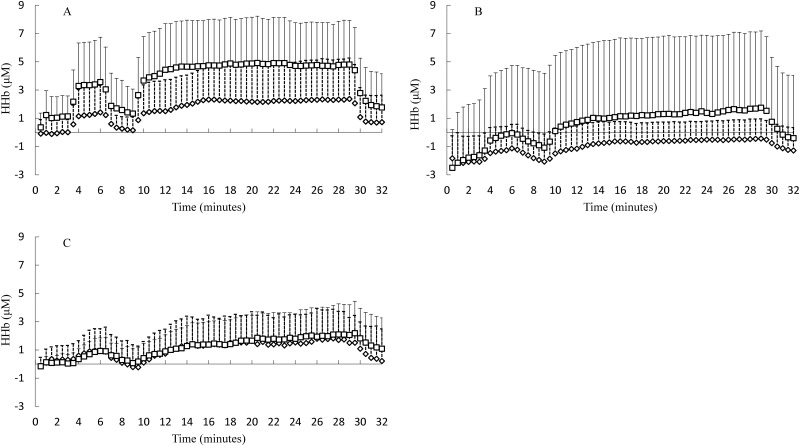
Square-Wave Constant Load (cycling); deoxygenated haemoglobin (HHb) measures. Panel (A) HHb in the LVL. Panel (B) HHb in the GAST. Panel (C) HHb in the PFC during the SW/CL.

**Fig 3:**
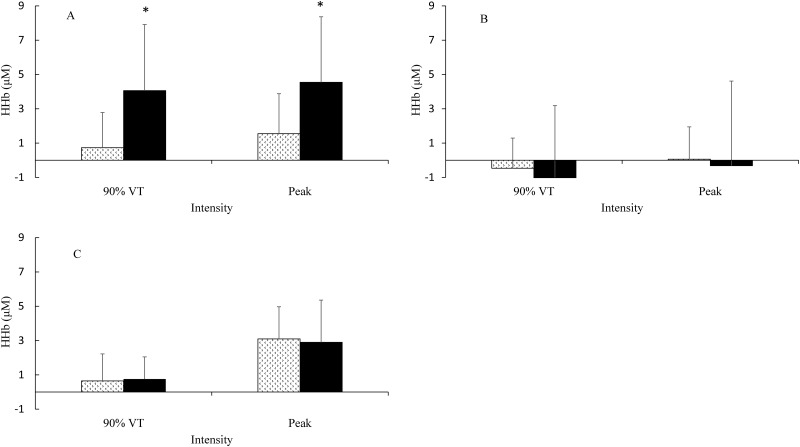
Ramp Incremental (cycling); deoxygenated haemoglobin (HHb) group mean measures. Panel (A) HHb in the LVL. Panel (B) HHb in the GAST. Panel (C) HHb in the PFC at 90% VTP and peak exercise. Pattern fill STT, solid fill LTT. * Significant different between groups.

**Fig 4:**
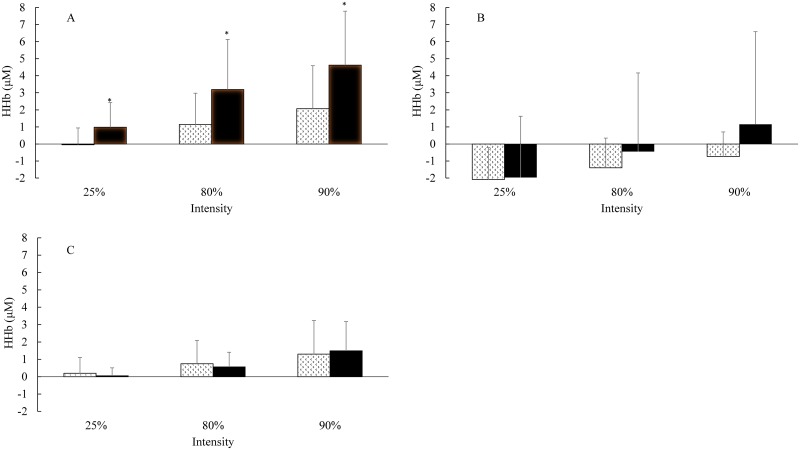
Square-Wave Constant Load (cycling); deoxygenated haemoglobin (HHb) group mean measures. Panel (A) HHb in the LVL. Panel (B) HHb in the GAST. Panel (C) HHb in the PFC at 90% VTP and peak exercise. Pattern fill STT, solid fill LTT. * Significant different between groups.

All NIRS-derived raw data (O_2_Hb, HHb, tHb and TSI) were recorded at 10 Hz. The last 20 seconds of resting values were averaged to obtain baseline values. All changes were then expressed relative to these baseline values, then calculated and displayed as 30 second (Figs 1 & 2) and total data for each intensity (Figs 3 and 4) averages for RI and SWCL respectively. Compared to O_2_Hb, HHb is less affected by changes in blood haemodynamics [45–47], thus a better indicator of oxygenation. Further, in studies similar to the current study use HHb is used when describing changes in O_2_ utilisation, [8, 23, 48] therefore, O_2_Hb data is not presented for the current study. The HHb results for the RVL were the same as the LVL for the RI and SWCL, providing additional evidence that the NIRS was providing consistent HHb data, and therefore, to avoid providing similar data, the RVL data have not been presented.

### Statistical Analysis

All statistical analyses were performed using SPSS (version 22, SPSS Inc., Chicago, IL). A second investigator checked all data for correct input. Prior to statistical analysis data were checked for normality and that relevant assumptions were met. To identify the presence of any significant exercise intensity and group (STT vs LTT) main effects as well as interactions while controlling for any between group total weekly training load differences, two-way (group: STT and LTT, by intensity: 90% VTP and peak, and 25% [first bout at 25%], 80% and 90% VTP) ANCOVAs (Analysis of Covariance’s) were conducted on each of the dependent variables and for each of the conditions. Following significant main effects post hoc tests were not required as they were on two levels. For significant interactions, post hoc pairwise t-tests were conducted. For all analysis, significance was set at the 95% level of confidence. Partial-eta squared was used to determine the effect size of small (ɳ_Ƥ_^2^ = 0.01) medium (ɳ_Ƥ_^2^ = 0.06) or large (ɳ_Ƥ_^2^ = 0.14) was determined as per Cohen [49].

## Results

### Systemic Oxygen Utilisation

For relative VO_2_ (mL^.^ kg^-1.^ min^-1^) in the RI, there was a significant group main effect with a large effect size [F(1, 23) = 13.987; p = 0.001; ɳ_Ƥ_^2^ = 0.378; β = 0.947], with VO_2_ higher in LTT. There was a significant main effect with a large effect for intensity [F(1, 23) = 40.359; p < 0.001; ɳ_Ƥ_^2^ = 0.637; β = 0.000], with VO_2_ higher in LTT. There was no significant group x intensity interaction [F (1, 23), = 3.317; p = 0.082; ɳ_Ƥ_^2^ = 0.126; β = 0.415] (Table 2).

**Table 2:** Ramp Incremental (cycling); systemic oxygen utilisation, ventilatory and heart rate measures.

	90% TP		Peak	
	STT	LTT	STT	LTT
VO_2_ (mL^.^ kg^-1.^ min^-1^)	20.2 (5.1)	29.0 (6.4) *	30.4 (5.8)	40.8 (7.1) *
VO_2_ (L^.^ min^-1^)	1.4 (0.4)	1.8 (0.3) *	2.1 (0.4)	2.6 (0.4) *
V_E_ (L^.^ min^-1^)	34.9 (10.4)	47.0 (8.5) *	79.6 (21.9)	102.1(16.2)*
V_T_ (L)	1.5 (0.3)	2.0 (0.2) *	1.9 (0.4)	2.1 (0.2) *
BF (Breaths^.^ min^-1^)	24.1 (5.5)	23.7 (3.9)	43.2 (10.0)	48.9 (10.4
HR (Beats^.^ min^-1^)#	123.8 (16.9)	134.4 (7.5)	160.3 (10.8)	168.0 (10.8)

Values are mean (SD).

* Significant difference between groups p = < 0.05.

VTP: ventilatory turn point; STT: Short Term Trained; LTT: Long Term Trained; VO_2_: oxygen utilisation; VE: minute ventilation; V_T_ tidal volume; BF: breathing frequency; HR: heart rate.

^#^HR STT n = 10, LTT n = 9.

For absolute VO_2_ (L^.^ min^-1^) in the RI, there was a significant group main effect with a medium effect size [F(1, 23) = 7.775; p = 0.010; ɳ_Ƥ_^2^ = 0.253; β = 0.761], with VO_2_ higher in LTT. There was a significant main effect with a large effect for intensity [F(1, 23) = 18.542; p < 0.001; ɳ_Ƥ_^2^ = 0.446; β = 0.985], with VO_2_ higher in LTT. There was no significant group x intensity interaction [F (1, 23), = 0.262; p = 0.614; ɳ_Ƥ_^2^ = 0.011; β = 0.078] (Table 2).

For relative VO_2_ (mL^.^ kg^-1.^ min^-1^) in the SWCL, there was a significant group main effect with a large effect size [F(1, 23) = 13.358; p = 0.001; ɳ_Ƥ_^2^ = 0.367; β = 0.938], with VO_2_ higher the LTT. There was a significant main effect with a large effect size for intensity [F(2, 46) = 23.552; p < 0.001; ɳ_Ƥ_^2^ = 0.506; β = 1.000], with VO_2_ higher in LTT. There was a significant group x intensity interaction [F(2, 46), = 11.627; p < 0.001; ɳ_Ƥ_^2^ = 0.336; β = 0.991], with VO_2_ being higher in LTT compared to the STT at 25%, 80% and 90% VTP (Table 3).

**Table 3:** Square–Wave Constant Load (cycling); systemic oxygen utilisation, ventilatory, heart rate and rate of perceived exertion measures.

	25% TP		80% TP		90% TP	
	STT	LTT	STT	LTT	STT	LTT
VO_2_ (mL^.^ kg^-1.^ min^-1^)	9.0 (1.5)	11.7 (2.4) *	17.9 (3.0) *	23.2 (4.7) *	23.2 (4.8)	31.6 (5.5) *
VO_2_ (L · min^-1^)	0.6 (0.1)	0.7 (0.1) *	1.3 (0.2)	1.5 (0.3) *	1.6 (0.3)	2.0 (0.4) *
V_E_ (L · min^-1^)	16.8 (2.5)	19.7 (4.3) *	31.3 (5.8)	37.7 (7.4) *	46.9 (9.5)	59.3 (10.0) *
V_T_ (L)	0.9 (0.1)	1.0 (0.2)	1.4 (0.2)	1.6 (0.2) *	1.6 (0.2)	2.0 (0.3) *
BF (Breaths^.^ min^-1^)	18.7 (3.4)	20.1 (3.2)	21.6 (3.3)	23.4 (3.3)	28.4 (4.4)	30.5 (3.5)
HR (Beats^.^ min^-1^)^#^	87.1 (5.6)	85.4 (6.7)	115.6 (10.4)	115.6 (8.6)	139.9 (15.4)	145.2 (10.3)
RPE	1.00 (00)	1.00 (0.00)	2.9 (0.6)	3.0 (0.7)	7.1 (1.2)	8.1 (1.1)

Values are mean (SD).

* Significant difference between groups p = < 0.05.

STT: Short Term Trained; LTT: Long Term Trained; VO_2_: oxygen utilisation: VE: minute ventilation; V_T_ tidal volume; BF: breathing frequency; HR: heart rate (BPM: beats per minute).

^#^HR STT n = 11, LTT n = 13.

For absolute VO_2_ (L^.^ min^-1^) in the SWCL, there was a significant group main effect with a large effect size [F(1, 23) = 4.776; p = 0.039; ɳ_Ƥ_^2^ = 0.172; β = 0.553], with VO_2_ higher the LTT. There was a significant main effect with a large effect size for intensity [F(2, 46) = 17.907; p < 0.001; ɳ_Ƥ_^2^ = 0.438; β = 1.000], with VO_2_ higher in LTT. There was a significant group x intensity interaction [F(2, 46), = 4.937; p = 0.011; ɳ_Ƥ_^2^ = 0.177; β = 0.782], with VO_2_ being higher in LTT compared to the STT at 25%, 80% and 90% VTP by 0.1 L^.^ min^-1^, 0.2 L^.^ min^-1^ and 0.4 L^.^ min^-1^ respectively, and the difference between the groups widening as the intensity increased (Table 3).

### Ventilation

#### Minute Ventilation

For V_E_ in the RI, there was a significant group main effect with a large effect size [F(1, 23) = 8.630; p = 0.007; ɳ_Ƥ_^2^ = 0.273; β = 0.803], with V_E_ being higher in LTT. There was a significant main effect for intensity with a large effect size [F(1, 23) = 20.281; p < 0.001; ɳ_Ƥ_^2^ = 0.469; β = 0.991], with V_E_ being higher in LTT. There was no significant group x intensity interaction [F(1, 23) = 3.495; p = 0.074; ɳ_Ƥ_^2^ = 0.132; β = 0.433] (Table 2).

For V_E_ in the SWCL, there was a significant group main effect with a large effect size [F(1, 23] = 8.830; p = 0.007; ɳ_Ƥ_^2^ = 0.277; β = 0.812), with V_E_ being higher in LTT. There was a significant main effect for intensity with a large effect size [F(2, 46) = 11.640; p < 0.001; ɳ_Ƥ_^2^ = 0.336; β = 0.991], with V_E_ being higher in LTT. There was a significant group x intensity interaction [F(2, 46) = 7.096; p = 0.002; ɳ_Ƥ_^2^ = 0.236; β = 0.914], with V_E_ being higher in LTT compared to the STT at 25% 80% and 90% VTP, with the difference between groups increasing as intensity increased (Table 3).

#### Tidal Volume

For V_T_ in the RI, there was a significant group main effect with a large effect size [F(1, 23) = 11.958; p = 0.002; ɳ_Ƥ_^2^ = 0.342; β = 0.912], with V_T_ being higher in LTT. There was no significant main effect for intensity [F(1, 23) = 0.422; p = 0.522; ɳ_Ƥ_^2^ = 0.018; β = 0.096]. There was a significant group x intensity interaction [F(1, 23) = 7.929; p = 0.010; ɳ_Ƥ_^2^ = 0.256; β = 0.769], with V_T_ being higher in LTT compared to the STT at 90% VTP, with the difference between groups decreased from 90% TP to peak exercise (Table 2).

For V_T_ the SWCL, there was a significant group main effect with a large effect size [F(1, 23) = 6.471; p = 0.018; ɳ_Ƥ_^2^ = 0.220; β = 0.683], with V_T_ being higher in LTT. There was a significant main effect for intensity with a large effect size [F(2, 46) = 16.475; p < 0.001; ɳ_Ƥ_^2^ = 0.417; β = 0.999], with V_T_ being higher in LTT. There was a significant group x intensity interaction [F(2, 46) = 6.205; p = 0.004; ɳ_Ƥ_^2^ = 0.212; β = 0.872], with V_E_ being higher in LTT compared to the STT at 25% 80% and 90% VTP, with the difference between groups widening as intensity increased (Table 3).

#### Breathing Frequency

For BF in the RI, there was no significant group main effect [F(1, 23) = 0.851; p = 0.366; ɳ_Ƥ_^2^ = 0.036; β = 0.143]. There was a significant main effect for intensity with a large effect size [F(1, 23) = 17.144; p < 0.001; ɳ_Ƥ_^2^ = 0.427; β = 0.977], with BF being higher in LTT. There was no significant group x intensity interaction [F(1, 23) = 4.235; p = 0.051; ɳ_Ƥ_^2^ = 0.155; β = 0.505] (Table 2).

For BF in the SWCL, there was no significant group main effect [F(1, 23) = 1.173; p = 0.290; ɳ_Ƥ_^2^ = 0.049; β = 0.180]. There was a significant main effect for intensity with a large effect size [F(2, 46) = 4.004; p = 0.025; ɳ_Ƥ_^2^ = 0.148; β = 0.688], with BF being higher in LTT. There was no significant group x intensity interaction [F(2, 46) = 0.043; p = 0.958; ɳ_Ƥ_^2^ = 0.002; β = 0.056] (Table 3).

#### Heart Rate

For HR in the RI, there was no significant group main effect [F(1, 21) = 3.993; p = 0.059; ɳ_Ƥ_^2^ = 0.160; β = 0.479]. There was a significant main effect for intensity with a large effect size [F(1, 21) = 71.280; p < 0.001; ɳ_Ƥ_^2^ = 0.772; β = 1.000], with HR being higher in LTT. There was no significant group x intensity interaction [F(1, 21) = 0.802; p = 0.381; ɳ_Ƥ_^2^ = 0.037; β = 0.137] (Table 2).

For HR in the SWCL, there was no significant group main effect [F(1, 14) = 0.128; p = 0.726; ɳ_Ƥ_^2^ = 0.009; β = 0.063]. There was a significant main effect for intensity with a large effect size [F(2, 28) = 35.041; p < 0.001; ɳ_Ƥ_^2^ = 0.715; β = 1.000], with HR being higher in STT. There was no significant group x intensity interaction [F(2, 28) = 1.134; p = 0.336; ɳ_Ƥ_^2^ = 0.075; β = 0.229] (Table 3).

#### Rating of Perceived Exertion

For RPE in the SWCL, there was no significant group main effect [F(1, 23) = 2.882; p = 0.103; ɳ_Ƥ_^2^ = 0.111; β = 0.370]. There was a significant main effect for intensity with a large effect size [F(2, 46) = 23.712; p < 0.001; ɳ_Ƥ_^2^ = 0.505; β = 1.000], with RPE being higher in LTT. There was no group x intensity interaction [F(2, 46) = 2.969; p = 0.078; ɳ_Ƥ_^2^ = 0.105; β = 0.5.8] (Table 3).

### Tissue Deoxyhaemoglobin

The HHb changes in the VL, GAST and PFC in RI followed the expected pattern (Fig 1). The HHb changes in the VL, GAST and PFC in SWCL followed the expected pattern (Fig 2).

#### Left Vastus Lateralis

For LVL HHb in the RI, there was a significant group main effect for with a large effect size [F(1, 23) = 7.176; p = 0.013; ɳ_Ƥ_^2^ = 0.238; β = 0.728], with HHb being higher in LTT. There was a significant main effect for intensity with a large effect size [F(1, 23) = 8.844; p = 0.007; ɳ_Ƥ_^2^ = 0.278; β = 0.813], with HHb being higher in LTT. There was no significant group x intensity interaction [F(1, 23) = 0.088; p = 0.770; ɳ_Ƥ_^2^ = 0.004; β = 0.059] (Fig 3).

For LVL HHb in the SWCL, there was a significant group main effect with a large effect size [F(1, 23) = 5.243; p = 0.032; ɳ_Ƥ_^2^ = 0.186; β = 0.592], with HHb being higher in LTT. There was a significant main effect for intensity with a large effect size [F(2, 46) = 3.884; p = 0.028; ɳ_Ƥ_^2^ = 0.144; β = 0.673], with HHb being higher in LTT. There was a significant group x intensity interaction [F(2, 46) = 3.431; p = 0.041; ɳ_Ƥ_^2^ = 0.130; β = 0.616], with HHb being higher in LTT compared to the STT at 25%, 80% and 90% VTP, with the difference between groups increasing as intensity increased (Fig 4).

#### Gastrocnemius

For the GAST in the RI, there was no significant group main effect [F(1, 21) = 0.052; p = 0.821; ɳ_Ƥ_^2^ = 0.002; β = 0.056]. There was no significant main effect for intensity [F(1, 21) = 0.399; p = 0.534; ɳ_Ƥ_^2^ = 0.017; β = 0.093]. There was no group x intensity interaction [F(1, 21) = 0.131; p = 0.720; ɳ_Ƥ_^2^ = 0.006; β = 0.064] (Fig 3).

For the GAST in the SWCL, there was no significant group main effect [F(1, 21) = 0.005; p = 0.944; ɳ_Ƥ_^2^ = 0.000; β = 0.051]. There was no significant main effect for intensity [F(2, 42) = 0.041; p = 0.960; ɳ_Ƥ_^2^ = 0.002; β = 0.056]. There was no group x intensity interaction [F(2, 42) = 2.277; p = 0.115; ɳ_Ƥ_^2^ = 0.098; β = 0.437] (Fig 4).

#### Pre-Frontal Cortex

For PFC HHb in the RI, there was no significant group main effect [F(1, 23) = 0.033; p = 0.857; ɳ_Ƥ_^2^ = 0.001; β = 0.054]. There was a significant main effect for intensity with a large effect size [F(1, 23) = 6.367; p = 0.019; ɳ_Ƥ_^2^ = 0.217; β = 0.676], with HHb being higher in LTT. There was no group x intensity interaction [F(1, 23) = 0.085; p = 0.773; ɳ_Ƥ_^2^ = 0.004; β = 0.059] (Fig 3).

For PFC HHb in the SWCL, there was no significant group main effect [F(1, 23) = 0.000; p = 0.999; ɳ_Ƥ_^2^ = 0.000; β = 0.050]. There was no significant main effect for intensity [F(2, 46) = 0.840; p = 0.438; ɳ_Ƥ_^2^ = 0.035; β = 0.185]. There was no group x intensity interaction [F(2, 46) = 0.239; p = 0.788; ɳ_Ƥ_^2^ = 0.010; β = 0.085] (Fig 4).

## Discussion

This current study is unique in that it describes VO_2_, and multiple local muscle and PFC HHb during exercise in older women closely matched for current training load. It was hypothesised that VO_2_ and HHb would be higher in LTT compared to the STT at peak exercise, but similar when exercising at 25%, 80% and 90% VTP. The VO_2_ was significantly higher in LTT compared to the STT at peak exercise and at 25%, 80% and 90% VTP. Deoxyhaemoglobin (HHb) in the LVL was also significantly higher in LTT compared to the STT at peak exercise and at 25%, 80% and 90% VTP in the LV only. No difference in HHb was found for GAST or PFC at any intensity. A primary factor influencing VO_2_ and HHb during exercise is current training status, with greater VO_2_ and HHb in higher trained compared to less trained counterparts [1, 2, 21]. Therefore, the current study ensured that current training load between STT and LTT was closely matched, with the average difference between LTT and STT participants being nine minutes of moderate intensity exercise per day.

### Systemic Oxygen Utilisation

This current study supported the hypothesis that, when matched for current training load, LTT older women have a significantly higher VO_2peak_ than STT older women. The absolute VO_2peak_ values of 2.6 ± 0.4 L · min^-1^ for the LTT and 2.1 ± 0.4 L · min^-1^ for the STT are higher than reference values for untrained women of this age [50], therefore, are consistent with the training volume and experience reported by the participants. These results of the current study support others [12, 51, 52] in that VO_2peak_ could be the primary physiological mechanism reflective of performance in older adults. However, they are in contrast to a dose response relationship existing between ET and improved VO_2peak_ [1], and evidence that maximum gains in VO_2peak_ are achieved within 12 months of ET [2, 53]. Our results suggest there is potential for VO_2peak_ to increase beyond two years of regular ET. A possible reason for the conflict between the current VO_2peak_ measures and others is that other studies have not investigated the changes in VO_2peak_ beyond 12 months in older adults [53], rather based their findings on plateaus reported in a 12 month period. Another potential explanation is all training studies in older women have been on those aged over 60 and previously untrained, therefore, training intensity and volume might not have been sufficient to elicit continued training adaptations [4, 8, 9].

As expected, absolute VTP values were significantly higher in the LLT compared to the STT, however, this difference was not seen when expressed as a percentage of VO_2peak_. This suggests that a higher VTP as a percentage of VO_2peak_ did not influence the higher VO_2peak_ in LTT compared to the STT. This supports Marcell et al. [54] who reported VTP as a percentage of VO_2peak_ is not a determining factor in aerobic exercise performance.

During the SWCL, contrary to our hypothesis, VO_2_ (L · min^-1^) was significantly higher in LTT compared to the STT while cycling at a Power calculated as 25%, 80% and 90% of that at VTP. Moreover, as the intensity increased the difference between the groups changed from 0.1 L · min^-1^ at 25%, to 0.2 L · min^-1^ 80% and 0.04 L · min^-1^ at 90% (as indicated by the ANCOVA interactions). These results support Dogra et al. [21] who reported higher VO_2_ values in trained compared to untrained older women during constant load cycling at 90% VTP. This suggests that while exercising at the same relative intensity, those who are longer trained have improved O_2_ utilisation during sub-VTP constant load exercise.

### Ventilation

An unexpected finding of this current study was the higher V_E_ in LTT at peak exercise and during the SWCL was produced by increased V_T_, not BF. These findings are in contrast to the response in typically healthy adults performing strenuous exercise, where V_T_ plateaus once it reaches ~ 50–60% of vital capacity and further increases in V_E_ result exclusively form increases in BF [14]. While increases in V_Emax_ following ET have been reported in older women [53, 55–57], this current study is unique in reporting the effect of short and long term ET on BF, V_E_ and V_T_ at peak and sub—VTP intensities. These results indicate that the higher V_E_ of the LTT was produced from breathing larger volumes of air (V_T_) rather than increases in BF. These higher volumes could be result of the LTT having more compliant connective tissue of the lungs and or better respiratory muscle function as a result of their additional years of training [58].

### Heart Rate and Rate of Perceived Exertion

As expected, peak HR was not different between groups during the RI. This supports other research that indicates that peak HR is a function of age, not training [30, 57]. A difference in the response of HR following transitions from light to moderate intensity steady state cycling could have implications for O_2_ delivery during SWCL exercise. Others have reported faster HR dynamics in trained compared to untrained older women, [4, 21], however, these adaptations can plateau after just nine weeks of regular training [4]. In the current study, HR was not different between groups during the SWCL, therefore, potentially had no influence on the results of the other dependent variables.

The rating of perceived exertion provides an additional valid method of evaluating effort based on subjective sensation [59]. The SWCL intensities were calculated as a percentage of VTP, which could potentially have a subjective component; however, RPE was not different between groups during the SWCL, indicating that both groups were exercising at the same relative intensity.

### Tissue Deoxyhaemoglobin

A unique component of this study was the simultaneous measurement of deoxyhaemoglobin (HHb) in multiple skeletal muscle and PFC. The results supported the hypothesis in that significantly higher absolute HHb were observed in the VL in the LLT compared to the STT at 90% VTP and peak exercise. However, the significantly higher HHb would be observed in the VL in LTT at each relative SWCL intensity (25%, 80% and 90% of VTP) is contrary to the hypothesis. This suggests that regardless of exercise intensity, compared to STT, LTT utilise higher amounts of O_2_ in the VL. A further unique component of the current study was the simultaneous measurement of HHb in the RVL and LVL. Results indicated that both legs utilised the same amount of O_2_ during exercise, and that the devices were measuring consistently.

Previously, only three studies have investigated HHb in exercising muscles of older women. Two compared young with old; one was unable to provide data due to technical difficulties [23], and the other performed knee extension exercise [48]. The third [21] reported a miss-matching of O_2_ delivery and utilisation (relationship between the time constants of HHb and VO_2_) in the VL during moderate exercise. While a peak exercise test was conducted in order to calculate the sub-maximal intensity, the authors did not report HHb values during the peak test. However, the higher absolute changes in HHb in the trained compared to untrained group during sub—VTP constant load exercise support the current study.

Observing no difference in HHb between the groups at GAST during the RI and SWCL are contrary the hypothesis. Oxygenation in the GAST during exercise has only ever been reported on men during treadmill running [60]. The investigators compared the HHb pattern of the gastrocnemius and vastus lateralis during ramp incremental exercise, reporting differences in the pattern of HHb between the two muscles, however, no patterns, values or possible rationale were presented. In the current study, while the differences in the VL was not observed in the GAST, the patterns were opposite. That is, while HHb gradually increased with intensity in the VL, in the GAST, there was a large drop in HHb at the onset of exercise which progressively decreased (less HHb) as exercise intensity increased.

Differences in fibre structure between the GAST and VL may help explain the differences in HHb response between the muscles. That is, compared to the VL, the GAST has a higher percentages of Type 1 fibres which during exercise have a high perfusion pressure and rate of O_2_ extraction, and lower percentages of Type IIa and IIb fibres which have a low perfusion pressure and rate O_2_ extraction [61, 62]. Further a reduction in muscle mass in older women has been associated with a concomitant reduction in leg blood flow and perfusion pressure [63]. Age-related reductions in oxidative capacity in the VL have not been reported in either sex, however, reductions have been reported in the GAST of men [64]. If this were the same in older women, it would help explain any differences in the recruitment and oxygen utilisation of the GAST compared to the VL during exercise.

Our observation of no difference in HHb between the STT and LTT groups in the PFC during the RI and SWCL support the hypothesis that the difference in training years between the groups in the current study would not influence PFC during exercise. The current study is unique in measuring the effect of ET on PFC HHb in healthy women, and supports similar findings in men [31]. Further, no differences have been reported in PFC HHb between trained men and women at peak exercise [30]. Therefore, it appears that training years does not affect PFC HHb in older women, thus is unlikely to contribute to limiting exercise capacity or performance in this population.

### Combined Responses

The higher VO_2peak_ values of the LTT compared to the STT indicate that improvements in the integration of the pulmonary, cardiovascular and muscular systems to uptake, transport and utilise O_2_, do not plateau after 12 months of aerobic ET as has been suggested [2, 53]. During exercise, the cascade of events to supply the exercising muscles with additional O_2_ to maintain metabolic processes begins with increased pulmonary ventilation. A full description of the processes involved in ventilation and respiratory mechanics is beyond the scope of this current study, however, up to a point increases in V_E_ result from increases in either V_T_ or BF, or both [65], where further increases in V_E_ result solely from increases in BF [14]. In contrast, this current study reported the higher V_E_ in LTT resulted solely from increase in V_T_ not BF. The effects of age and ET on respiratory control of older women is relatively unknown, with interventional studies required before suggesting a relationship exists. However, the current study supports the proposal that higher levels of physical fitness in older women might alter typical age-related increases in ΔV_E_ / ΔVCO_2_ [66].

Cardiac output (stroke volume x HR) provides the pressure and flow for oxygenated blood to reach exercising muscles for peripheral O_2_ extraction. As stroke volume and cardiac output were not measured in the current study, it is not possible to determine any influence on the higher VO_2peak_ of the LTT. However, as HR was not different between groups at any intensity during both conditions, and as maximum HR is not trainable, this indicates both groups were closely matched, and were exercising at the same relative intensity during the SWCL.

The higher HHb in the VL in LTT in this current study support others who have reported improved peripheral muscle O_2_ utilisation as a wider a-vO_2_diff in older women following ET [4–6]. This could in part be the result of improved capillary density in LTT, as invasive studies have reported increased capillarisation in older adults following ET [67–69]. While it is plausible to consider the higher HHb in the VL in LTT could be a function of and associated with the higher VO_2_, below VT, lactate and metabolic needs plateau [70], thus, additional O_2_ uptake at this intensity is unlikely to influence peripheral HHb. Further, hyperoxia during exercise increases VO_2_ and arterial O_2_ saturation, while muscle HHb is not effected [25], even at peak exercise [71]. Moreover, if peripheral tissue HHb were predominately influenced by VO_2_, the current study would have potentially observed significantly higher HHb in the GAST.

Monitoring of blood flow and muscle activation between the VL and GAST require further investigation, and would assist in determining the reasons for the differences in deoxyhaemoglobin patterns between muscles.

### Limitations

One potential limitation of this current study was the difference in the average age of the two groups, with the average age of the STT being four years older than the LTT, however, there was no group difference in peak HR, which is, predominately determined by age, providing support for the groups being well matched. A second limitation could be related to the average training load of LTT being higher than the STT. This was controlled for as best as possible through the recruiting process, and when tested was not significantly different. Nonetheless average weekly training loads were factored into the analysis as a covariant. A third limitation was that adipose tissue thickness was higher in STT compared to LTT. However, while high levels of adipose tissue may affect NIRS measures, all participants were within the recommended ranges (< 34 mm).

### Implications and Future Research

This current study has implications for endurance trained older women, in that beyond the typical systemic and peripheral adaptations that accompany initial regular ET, prolonged ET provides further positive systemic and intramuscular adaptations. Future research should include training studies of more than 12 months duration and include measures of the systemic, central, and peripheral components of O_2_ utilisation during and following prolonged ET.

## Conclusion

It is concluded that that in women aged 40–60 years, continued regular ET beyond two years can significantly improve VO_2_ at peak and constant load sub—VTP exercise. Further, these adaptations are concomitant with improvements in HHb in the VL during peak exercise and SWCL exercise below VTP. However, this higher deoxyhaemoglobin pattern is not observed in the GAST or PFC at any exercise intensity.
